# Pre-sternal thyroid swellings: a case of rare aberrant site recurrence and review of literature

**DOI:** 10.1186/s13044-019-0073-1

**Published:** 2019-11-21

**Authors:** Lovenish Bains, Sushant Bhatia, Rohit Kaushik, Sudhir Kumar Jain, Chandra Bhushan Singh, Shramana Mandal, Daljit Kaur

**Affiliations:** 1 Department of Surgery, Maulana Azad Medical College, New Delhi, India; 2 Department of Pathology, Maulana Azad Medical College, New Delhi, India; 3 Department of Transfusion Medicine, All India Institute of Medical Sciences, Rishikesh, India

**Keywords:** Pre-sternal, Metastasis, Recurrence, Papillary thyroid carcinoma (PTC), Retrosternal, Substernal

## Abstract

**Background:**

Thyroid swellings enlarge caudally into the mediastinum behind the sternum. Pre-sternal swelling of thyroid origin is very rare. We present our case of pre-sternal thyroid swelling which was albeit a surprisingly rare site of papillary thyroid carcinoma recurrence and review of pre-sternal thyroid swellings reported till date.

**Case summary:**

A 60 year old female presented with a painless, progressive swelling on the anterior part of the chest for the past 2 years. A 15 cm × 8 cm vertically aligned, non tender, well defined swelling was present on the pre-sternal region, with consistency ranging from soft to firm. The swelling was fixed to the underlying tissues and a fixed level IV lymph node was palpable on the right side. Ultrasonography revealed a large mass of 15 × 7 cm with multiple cystic areas. Fine needle aspiration cytology was inconclusive twice. Patient had undergone a total thyroidectomy for papillary carcinoma 10 years back. Computed tomography findings revealed a large 15 × 6.6 × 7 cm lobulated, pre-sternal, soft tissue lesion with solid & cystic components. The mass was infiltrating the right sided strap muscles and sternocleidomastoid. FNAC was inconclusive and thyroid scan could not pick up any activity in the mass. Henceforth a PET scan was done that showed increased FDG uptake by the lesion and the level IV lymph node. The patient underwent wide excision of the mass with right functional neck dissection, along with removal with both sternal head of sternocleido-mastoid, the strap muscles and the surrounding fascia. Histopathology confirmed papillary thyroid carcinoma. Patient received post-operative radioactive iodine ablation and is healthy with no recurrence up to 30 months of follow up.

**Discussion:**

The mechanisms for pre-sternal thyroid swelling are not understood due to paucity of cases. The mechanisms proposed are invasion of strap muscles and cervical linea alba and tumor cells spread anterior to sternum, truly ectopic thyroid tissue, de novo carcinogenesis in the embryonal remnants like the thyro-thymic residues, sequestered thyroid tissue which grows later or migration of thyroid cells, incomplete clearance at the time of primary surgery or intraoperative seeding.

**Conclusion:**

Pre-sternal region masses of thyroid origin are very rare. A proper work up, suspicion for thyroid mass and array of tests will be required to come to a provisional diagnosis. Since the masses reported in literature were primarily malignant, any such mass may be treated on lines of malignancy with radical surgery.

## Introduction

Almost all the goiters that enlarge inferiorly go into anterior-superior mediastinum due to the attachment of pretracheal fascia to the superior aspect of the sternum. Due to variation in the definition of substernal goiter, the incidence has wide range from 2.6–21% of patients undergoing thyroidectomy [1, 2]. The most commonly used definition states substernal goitre as a thyroid mass that has 50% or more of its volume located below the thoracic inlet. Substernal goiters represent up to 7% of mediastinal tumors [3]. Substernal goiter are always behind the sternum, extension in front of the sternum is very rare with limited 8 cases reported in indexed literature till date. Papillary thyroid carcinoma (PTC) is the most common form of well-differentiated thyroid cancer with a favorable prognosis. Recurrence rates of papillary thyroid carcinoma are in the range of 14–30%, predominantly in the cervical region and distant metastasis is known mainly to lungs and bones [4]. No case of pre-sternal recurrence of papillary thyroid carcinoma has ever been reported to the best of our knowledge in indexed literature. We present our unique case of 60 year old female with pre-sternal recurrence ten years after thyroidectomy.

## Case report

**Fig. 1:**
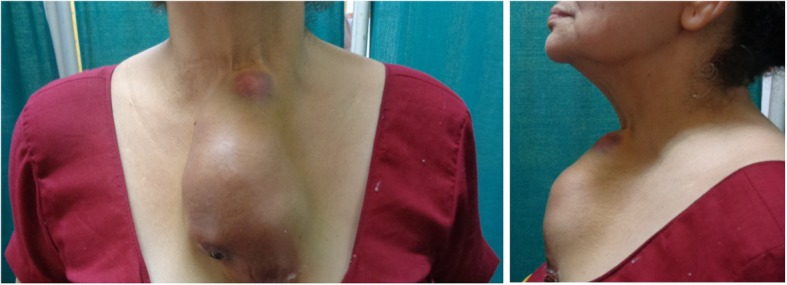
Pre-sternal swelling of 15 cm × 8 cm- anterior view & lateral view

**Fig. 2:**
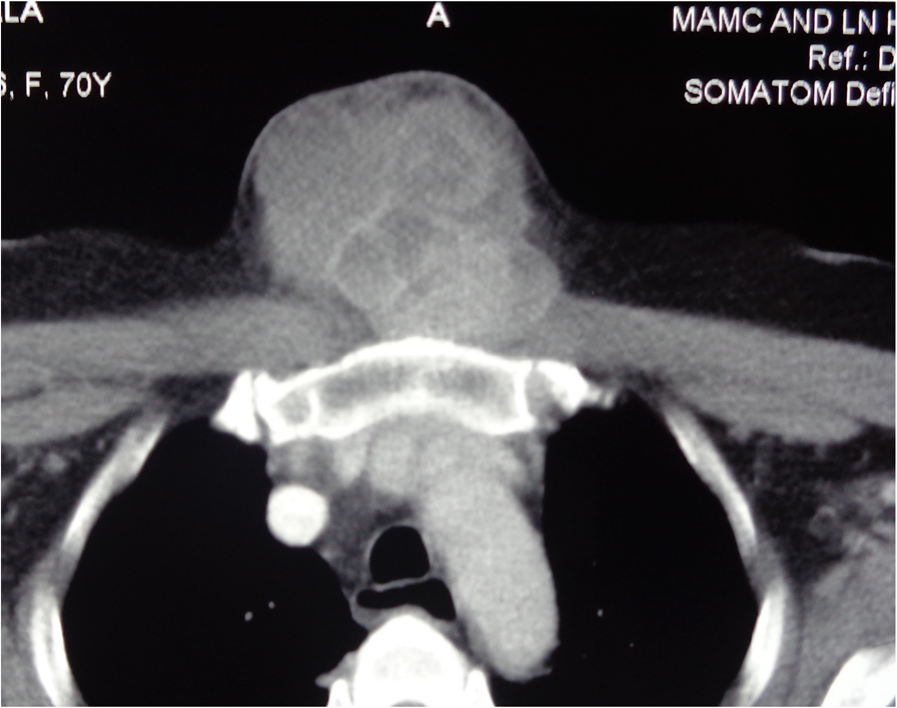
CT scan showing soft tissue mass anterior to sternum

**Fig. 3:**
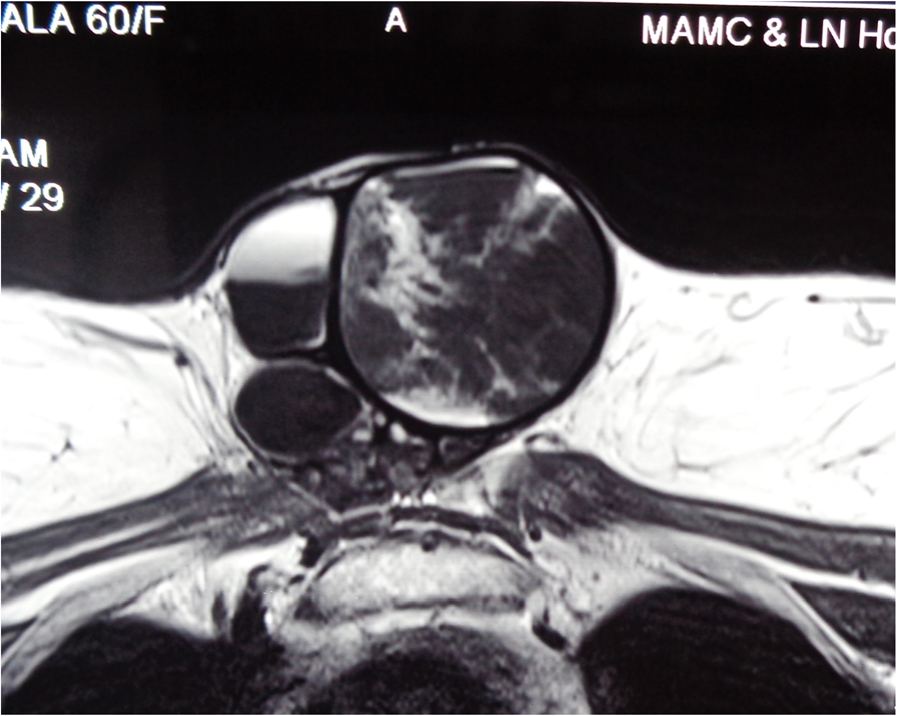
MRI coronal view showing large pre-sternal mass

**Fig. 4:**
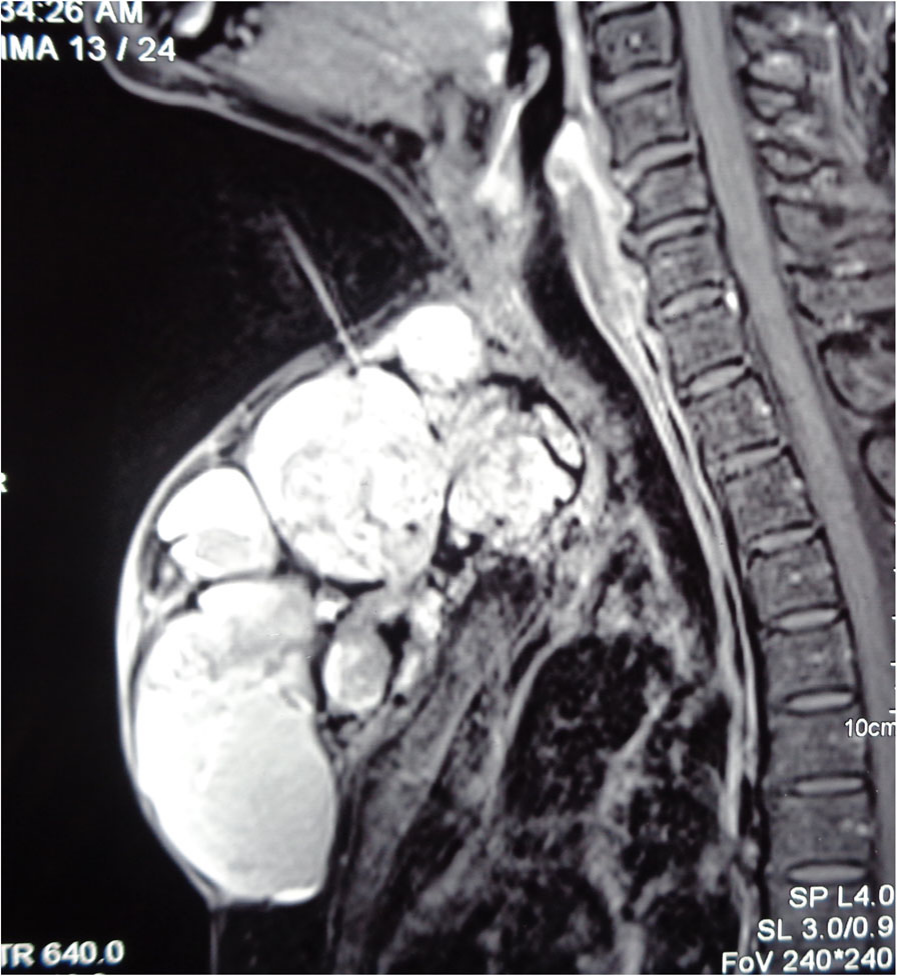
MRI sagittal view- multicystic lesion

A 60 year old female presented to our outpatient department with a painless, progressive swelling on the anterior part of the chest for the past 2 years. On examination, pulse rate was 78/min, blood pressure 130/80 mmHg and respiratory rate was 14/min. A 15 cm × 8 cm vertically aligned, non-tender, well defined swelling was present on the pre-sternal region, with consistency ranging from soft to firm (Fig. 1). The swelling was fixed to the underlying tissues. There was no visible neck swelling and retrosternal extension of this swelling was not appreciated. A 3 cm × 2 cm, hard, fixed level IV lymph node was palpable on the right side. Ultrasonography (USG) revealed a large mass of 15 × 7 cm with multiple cystic areas. Fine needle aspiration cytology was inconclusive twice. Patient had undergone a total thyroidectomy for papillary carcinoma 10 years back. No records were available and there was no history of radio iodine ablation therapy. Patient was taking 100 micrograms thyroxine daily. Her thyroid function tests were normal (TSH 3.1 uIU/ml; refence range 0.35–5.50). Her hemoglobin was 11 g % and laboratory parameters including kidney function tests within normal range. Computed tomography findings revealed a large 15 × 6.6 × 7 cm lobulated, pre-sternal, soft tissue lesion with solid & cystic components (Fig. 2). No underlying bony destruction or retro sternal extension was seen. No cervical lymphadenopathy was seen besides a 3 × 2 cm level IV node. The mass was infiltrating the right sided strap muscles and sternocleidomastoid. In view of invasion, Magnetic resonance imaging was also done, which revealed a large 14 × 6.5 × 7 cm well defined, multicystic lesion with solid components in the anterior aspect of lower neck extending inferiorly in the pre-sternal region (Figs. 3 and 4). The lesion shows ill-defined planes with bilateral sternocleido-mastoid muscles and the strap muscles.

**Fig. 5:**
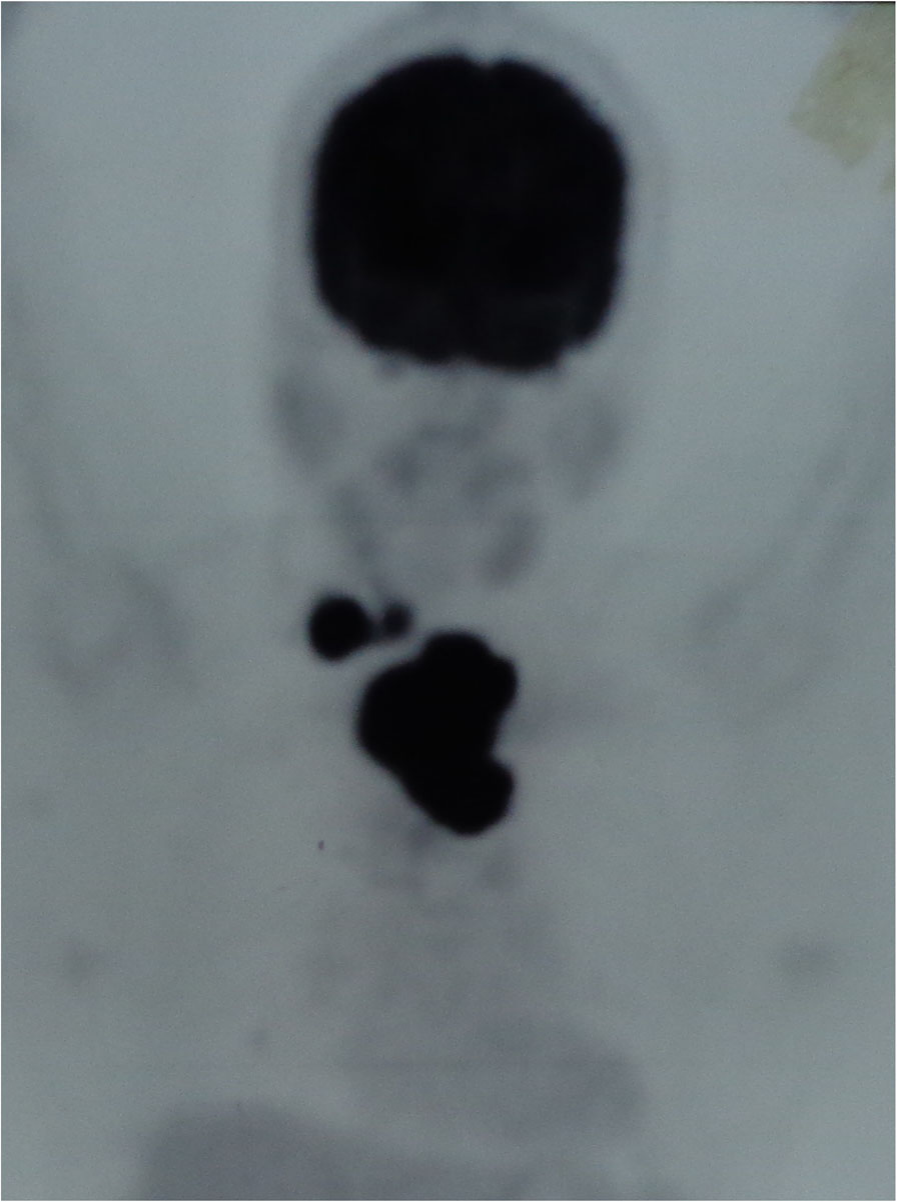
PET CT showing FDG uptake in the mass and neck node

**Fig. 6:**
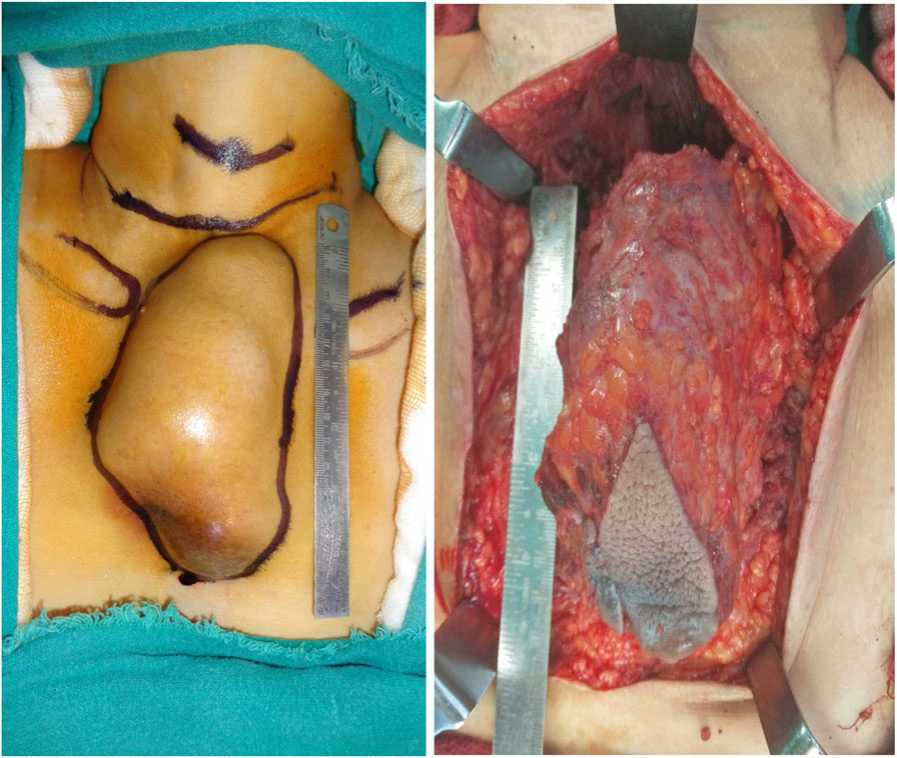
Pre-operative and intra-operative picture of the mass

**Fig. 7:**
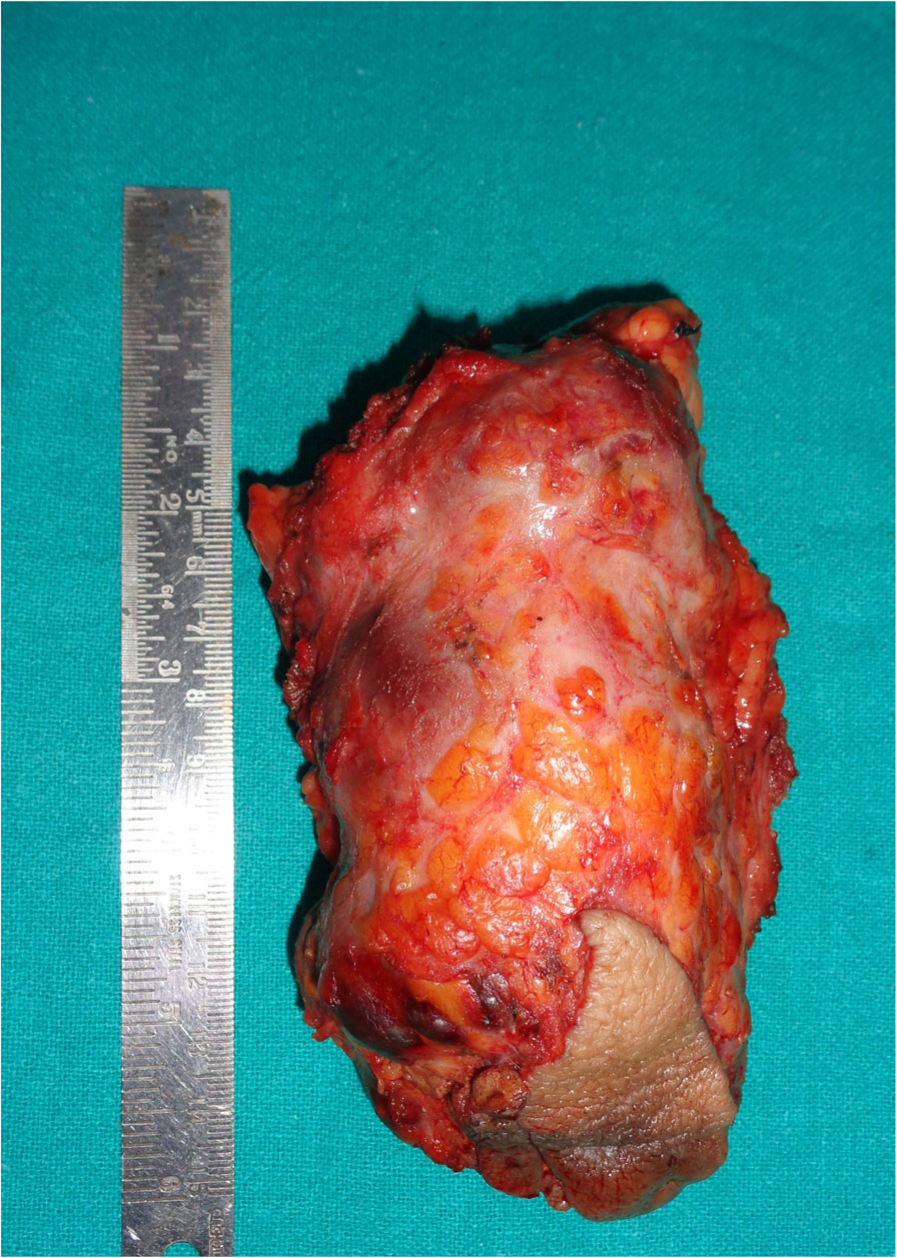
Resected specimen

A suspicion of recurrence of papillary thyroid cancer was thought in this rare location. Thyroid scan could not pick up any activity in the mass. Henceforth a PET scan was done that showed increased FDG uptake by the lesion and the level IV lymph node (Fig. 5). USG guided fine needle aspiration cytology was done again and showed Bethesda IV category. The patient underwent wide excision of the mass with right functional neck dissection. The mass was attached to the sternal head of the right sternocleido-mastoid and other strap muscles. The mass was removed with both sternal head of sternocleido-mastoid, the strap muscles and the surrounding fascia (Figs. 6 and 7).

**Fig. 8:**
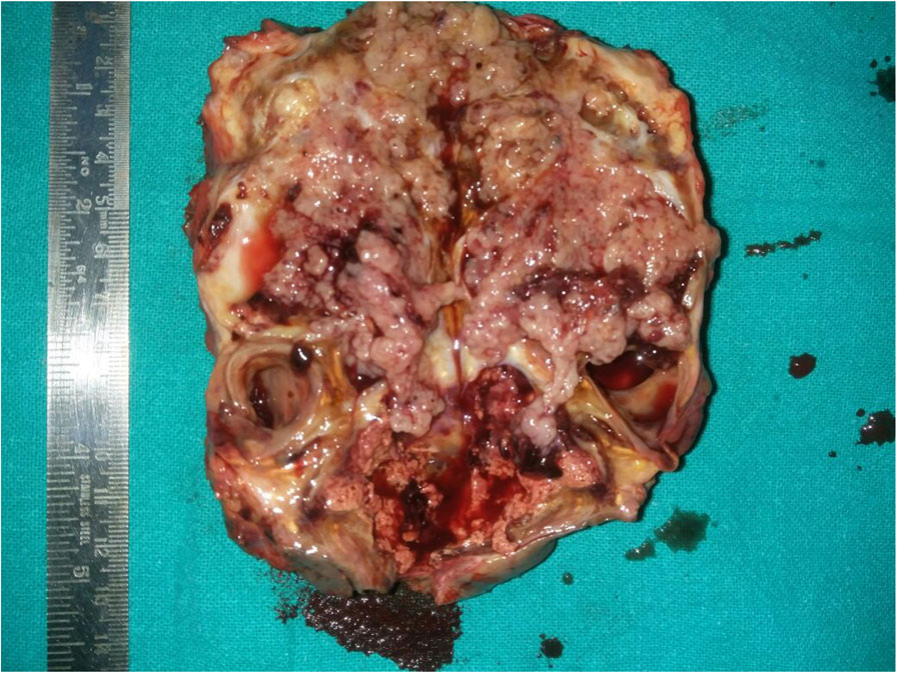
Specimen showing multiple cystic and solid areas

**Fig. 9:**
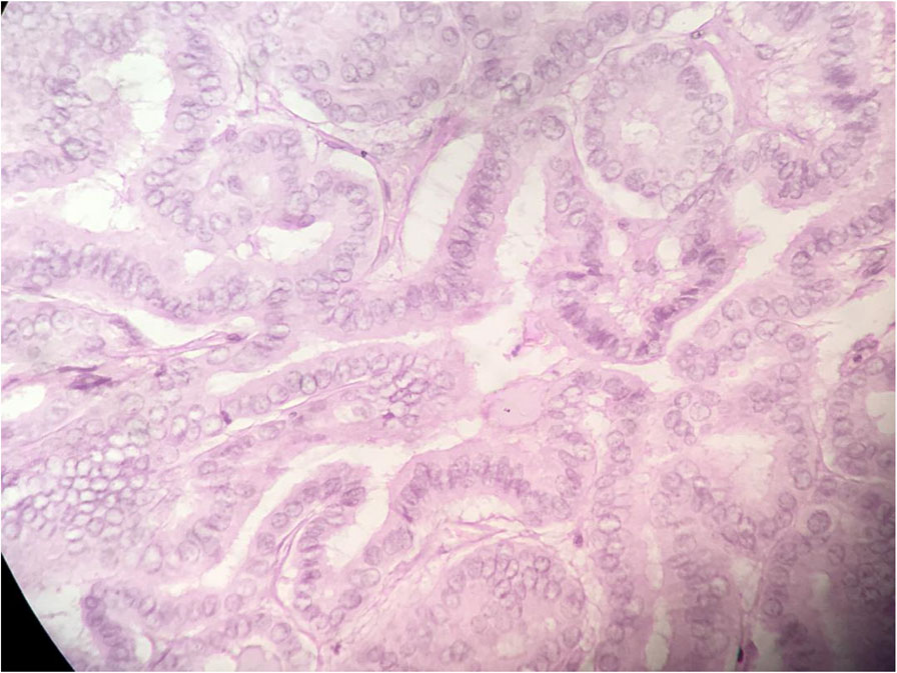
HPE consistent with papillary thyroid carcinoma. H & E stain

The gross specimen revealed solid components with multiple cystic areas (Fig. 8). Histopathology confirmed well differentiated papillary thyroid carcinoma with presence of typical cells, nuclear groves, inclusions and clustering. Focal area of necrosis and hemosiderin laden macrophages were also present (Fig. 9). Resected margins were clear. Labelled lymph node showed deposits of papillary carcinoma thyroid; included fibro collagenous tissue also showed deposits of papillary carcinoma thyroid. Post-operative radioactive iodine ablation 70 mCi was given approximately 8 weeks after the surgery. The thyroglobulin levels have been less than 1 microgram/L. The patient has been following regularly and is healthy with no recurrence up to 30 months of follow up.

## Discussion

Papillary thyroid carcinoma is the most common endocrine and thyroid cancer which accounts for 85% of all thyroid malignancies. Its incidence has been increasing worldwide as indicated by Surveillance, Epidemiology, and End Results (SEER) database from 1975 to 1992 when it increased from 4.8 to 5.8 per 100,000; however from 1992 to 2016, the incidence of papillary cancer has increased significantly from 5.8 to 14.1 per 100,000 [5].

Despite its well-differentiated characteristics, papillary carcinoma may be overtly or minimally invasive. In fact, these tumors may spread easily to other organs. Papillary carcinoma appears as an irregular solid or cystic mass or nodule in a normal thyroid parenchyma. Papillary tumors have a propensity to invade lymphatics but are less likely to invade blood vessels. These tumors are indolent and have a good prognosis, but frequently metastasize to regional lymph nodes in 5.4 to 13% of patients after initial surgery [6]. Recurrence rate after primary treatment is 1.8% annually for the first 10 years.

The 10-year, 20-year and 30-year recurrence rates were 11.3, 21.8 and 29.4%, respectively. The risk of recurrence of the disease is the greatest in the first 5 years (36.9%) after the initial treatment whereas 54.8% happened within the first 10 years [7]. Though the recurrence can occur any time, but maximum reported time was 43 years following total thyroidectomy and radioactive iodine ablation [8].

Local sites of recurrence include the cervical lymph nodes and the remaining thyroid tissue. Distant metastasis usually happens to bones and lungs, but less common sites include brain, adrenal, skeletal muscle [9]. Other rare sites have been liver, skin, parapharyngeal space and right atrium [10]. Risk factors implicated for loco regional recurrence are age, histological grade, distant metastases, extracapsular tumor extension, tumor size, and stage of disease [11].

Lymph node metastases are a debatable prognostic factor, having relevance in recurrence of disease, but have no relevance in survival rate of patients [12]. Recurrence increases with the number of metastatic nodes and the degree of metastasis, as well as with extracapsular infiltration and age [13]. This study also demonstrates that the presence of lymphatic permeation in the primary tumor correlates to an 84.4% probability of lymphatic metastasis. As with primary tumor size was of 3.8 cm versus 1.98 cm, only lymphatic permeation was correlated to an increase in metastasis in lymph nodes 65.4% versus 25% (*p* < 0.001). The metastatic frequency for cervical lymph node regions was IIa 18.5% versus 1.5%, III 24.3% versus 9.9%, IV 17.4% versus 18.1%, and VI 25.9% versus 71,2% [14].

The prognosis for PTC is better than for other types of thyroid cancer; however, the involvement of lymph nodes (LNs) is up to 15–30% at diagnosis [15]. The 5 year survival rate for local disease is near 100%, regional disease is near 100% and distant disease is 78% [16]. It is generally accepted that the spread pattern of LN in PTC is central compartment, ipsilateral compartment, and contralateral compartment sequentially. The factors that associated with contralateral LN metastasis were male gender, more than 2 cm size of main tumor, multifocality, bilaterality, and extra thyroidal extension.

Retrosternal goiter was first described by Haller in 1749 [17]. Substernal goiters are largely considered to result from the descent of a cervical goiter with the primary blood supply remaining in the neck, primarily from the inferior thyroid artery. Thereafter. thyroid gland enlarges into the retrosternal area owing its position beneath the pre-tracheal fascia and the strap muscles, all of which are attached to the top of manubrium.

Several factors favor the passage of the goiter into the mediastinum: Downward traction caused by normal swallowing, respiration creating negative intrathoracic pressure, and the pull of gravity on the goiter [18]. Some theories postulate that small cervical thyroid nodules descend beneath the pre-tracheal fascia, possibly aided by the negative intrathoracic pressures that normally occur during respiration and swallowing. As the nodule gradually enlarges, it eventually becomes trapped below the thoracic inlet, where it continues to enlarge within the confines of the thoracic cavity, commonly hidden from view and causes compressive effects as it enlarges. Malignancy can occur in 5–26.9% of substernal goiters [2].

By extensive search of literature we found only 8 reported cases (Table 1). These cases were essentially pre-sternal extension of the primary mass (multinodular goiter in one and papillary cancer in four) with heterotopic thyroid tissue in one and recurrence of colloid goiter in one. Details were not available for one case. Despite many cases of recurrence at unusual sites, no recurrence presenting as a pre-sternal mass has been reported. Pre-sternal thyroid swelling thus remains exceptionally rare presentation.

The mechanisms for pre-sternal presentation are not understood due to paucity of cases. The following mechanisms can be proposed.

**Table 1:** Details of pre-sternal thyroid swellings reported till date

S no	Author	Year	Age	Sex	Findings	Nature of mass
1	Belger S [23].	1952			Details not available	
2	Raman A et al. [21]	1999	39	F	15 × 30 cm Lobular cystic swelling on the anterior aspect of the neck from submental region to the xiphisternum in the pre-sternal plane	Papillary carcinoma thyroid with metastasis in regional lymph nodes
3	Brilli L et al. [24]	2007	50	M	10 cm in length Elongated neck mass, descending subcutaneously up to the mid region of the sternum.	Multinodular goiter
4	Chow TL et al. [19]	2014	50	M	3.5 × 3 cm dumb-bell–shaped mass, lower part in pre-sternal plane	Papillary carcinoma thyroid
5	Fanantenana HN et al. [20]	2015	60	M	18 × 14 cm Firm, smooth surface mass * H/o excision 7 years ago.	Heterotopic thyroid goitre with no evidence of malignancy.
6	Patil et al. [25]	2012	60	F	25 × 15 cm Mass arising from neck with multiple cysts in front of sternum	Papillary carcinoma thyroid
7	Tang et al. [26]	2015	42	M	17 × 13 cm Multiple cystic lesions in the chest beneath skin interlinked with the right thyroid lobe	Papillary thyroid microcarcinoma
8	Stumpf et al. [22]	2017	54	F	20 × 9 cm Large, firm, regular mass in pre-sternal region * H/o partial thyroidectomy 13 years ago.	Colloid goiter
9	Bains L et al	2019	60	F	15 × 8 cm	Papillary carcinoma thyroid recurrence

- invasion of strap muscles and cervical linea alba and tumor cells spread anterior to sternum [19]
- truly ectopic thyroid tissue [20]
- De novo carcinogenesis in the embryonal remnants like the thyro-thymic residues
- sequestered thyroid tissue which grows later or migration of thyroid cells [21]
- incomplete clearance at the time of primary surgery or intraoperative seeding [22]

## Conclusion

Pre sternal region masses of thyroid origin are exceptionally rare. A proper work up, suspicion for thyroid mass and array of tests will be required to come to a provisional diagnosis. Since the masses reported in literature were primarily malignant, any such mass may be treated on lines of malignancy with radical surgery.

## Data Availability

Not available.

## Abbreviations

CT: Computed tomography
FDG: Fluorodeoxyglucose
FNAC: Fine needle aspiration cytology
LN: Lymph node
MRI: Magnetic resonance imaging
PET: Positron emission tomography
PTC: Papillary thyroid carcinoma
USG: Ultrasonography
